# A drift-diffusion checkpoint model predicts a highly variable and growth-factor-sensitive portion of the cell cycle G1 phase

**DOI:** 10.1371/journal.pone.0192087

**Published:** 2018-02-12

**Authors:** Zack W. Jones, Rachel Leander, Vito Quaranta, Leonard A. Harris, Darren R. Tyson

**Affiliations:** 1 Department of Mathematical Sciences, Middle Tennessee State University, Murfreesboro, TN 37132, United States of America; 2 Department of Biochemistry, Vanderbilt University School of Medicine, Nashville, TN 37232, United States of America; Duke University, UNITED STATES

## Abstract

Even among isogenic cells, the time to progress through the cell cycle, or the intermitotic time (IMT), is highly variable. This variability has been a topic of research for several decades and numerous mathematical models have been proposed to explain it. Previously, we developed a top-down, stochastic drift-diffusion+threshold (DDT) model of a cell cycle checkpoint and showed that it can accurately describe experimentally-derived IMT distributions [Leander R, Allen EJ, Garbett SP, Tyson DR, Quaranta V. Derivation and experimental comparison of cell-division probability densities. J. Theor. Biol. 2014;358:129–135]. Here, we use the DDT modeling approach for both descriptive and predictive data analysis. We develop a custom numerical method for the reliable maximum likelihood estimation of model parameters in the absence of *a priori* knowledge about the number of detectable checkpoints. We employ this method to fit different variants of the DDT model (with one, two, and three checkpoints) to IMT data from multiple cell lines under different growth conditions and drug treatments. We find that a two-checkpoint model best describes the data, consistent with the notion that the cell cycle can be broadly separated into two steps: the commitment to divide and the process of cell division. The model predicts one part of the cell cycle to be highly variable and growth factor sensitive while the other is less variable and relatively refractory to growth factor signaling. Using experimental data that separates IMT into G1 vs. S, G2, and M phases, we show that the model-predicted growth-factor-sensitive part of the cell cycle corresponds to a portion of G1, consistent with previous studies suggesting that the commitment step is the primary source of IMT variability. These results demonstrate that a simple stochastic model, with just a handful of parameters, can provide fundamental insights into the biological underpinnings of cell cycle progression.

## Introduction

**Fig 1:**
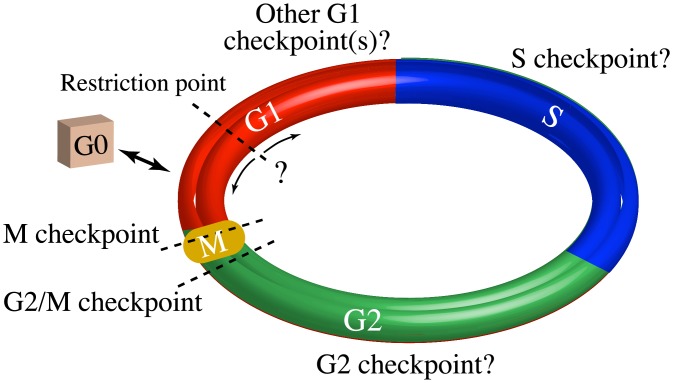
Simple illustration of the cell cycle. The four phases of the cell cycle (G1, S, G2, and M), the non-cycling G0 state, and three well-known checkpoints (dashed lines) are shown. The exact location and nature of the G1 checkpoint is controversial, indicated by ‘← ? →’. The number and location of other checkpoints within the G1, S, and G2 phases is also a topic of current research.

The process through which a cell replicates its DNA, doubles in size, and divides is known as the mitotic cell cycle [1] (Fig 1). The cell cycle proceeds unidirectionally: DNA synthesis (S phase) and the segregation of cellular components into two new daughter cells (mitosis or M phase) are separated by two “gap” phases (G1 and G2). The time it takes a cell to progress from the beginning of G1 to the end of M phase is referred to as the intermitotic time (IMT). Cell cycle progression is controlled by molecular signaling networks that verify the integrity of each step in this process; these verification points are referred to as checkpoints. Many distinct checkpoint functions have been described [2, 3], including checkpoints that assess: (i) growth factor signaling (often referred to as the restriction point [4]; see Fig 1); (ii) licensing of DNA replication to prevent reduplication [5]; (iii) nutrient abundance [6]; (iv) DNA damage [3]; (v) sufficient size of the cell prior to mitosis [7]; and (vi) proper machinery for chromosomal alignment and segregation during mitosis [8]. Hyperproliferative diseases, such as cancer, invariably suffer from defective cell cycle checkpoint function [2], usually caused by genetic mutations to important molecular regulators [9]. These mutations can disrupt the network structure in complex ways, reducing checkpoint fidelity and increasing IMT variability. An improved understanding of the molecular mechanisms underlying cell cycle checkpoints and IMT variability may thus lead to novel therapeutics that can restore normal cell function and/or slow or halt disease progression.

The origins and consequences of IMT variability have been the subject of intense research for decades [10–21]. For example, numerous papers have investigated the checkpoint in G1 that acts as the commitment step to cell division, often referred to as the restriction point. However, its position in the cell cycle, relationships to other G1 checkpoints, and the transition into and out of the non-cycling G0 state remain controversial [2, 4–6, 22–26]. In addition, how much of the variability in the total IMT is contributed before vs. after this step is a point of contention. Early studies by Zetterberg and Larsson suggest more variability occurs after the commitment step [22, 27], whereas others suggest that the variability arises prior to commitment [23, 24, 26]. Furthermore, although many of the important molecular components controlling checkpoint passage are known [2, 5, 28, 29], a comprehensive understanding of the complex network of molecular interactions that drives progression through the cell cycle is still lacking.

As a result, mathematical modeling has become an important tool for deriving new insights into cell cycle control and the origins of IMT variability [30–34]. Models describing IMT variability in mammalian cells include the Gaussian [35], gamma [36], log-normal [37], delayed exponential [15], exponentially-modified Gaussian (EMG) [38, 39], exponentially-modified gamma [40], convolutions of the above [41], and more complex distributions [42, 43]. For single-cell organisms, such as bacteria and yeast, a variety of ‘timer,’ ‘sizer,’ and ‘adder’ models, as well as mixed versions of these, have also been proposed [7, 31, 32, 44–46]. Whether applied to mammalian cells or single-cell organisms, a major drawback of these ‘top-down’ models is that the connection between IMT variability and the underlying biochemical processes driving cell cycle progression is often absent or unclear. Conversely, numerous ‘bottom-up’ models that explicitly represent the complex biochemical interactions that underlie cell cycle progression have been developed [34, 47, 48]. While these models can provide insights into, e.g., the relative contributions of extrinsic vs. intrinsic noise [34], their predictive ability is usually limited by the large numbers of uncertain parameters generally characteristic of such models [49]. The complexity of these models also precludes derivation of simple analytical expressions for the IMT distribution, limiting their ability to inform the stage- and phase-structured models that are typically used to study cell population-level behaviors [18, 50–52].

A simple, top-down model of the cell cycle that is both biologically informed and analytically tractable could thus be extremely useful in linking dynamic changes occurring at the molecular scale within individual cells to features of the population as a whole. In recent years, increasing evidence has emerged that cell fate decisions are driven by biochemical networks that act as ultra-sensitive switches [53]. For example, it has been shown that the commitment to apoptosis in higher eukaryotes occurs suddenly, in a “snap-action”-like manner, once pro-apoptotic proteins reach a critical threshold value [54]. Similarly, cells make an irreversible commitment to divide once pro-mitotic proteins obtain a critical threshold level [26, 33, 34]. Based on such observations, we recently proposed a model of checkpoint passage [55] in which a continuous random variable, assumed to be a function of one or more protein concentrations, evolves stochastically in time through a simple drift-diffusion process [56, 57]. Once the value of this variable reaches a critical threshold value, passage of the checkpoint is assumed to occur instantaneously. The time to checkpoint passage is thus translated into a first-exit time problem, which is analytically tractable [56, 57]. We refer to this model as the drift-diffusion+threshold (DDT) model.

Previously, we investigated the *descriptive* ability of the DDT modeling approach [55]. In this work, we investigate its *predictive* potential by fitting one-, two-, and three-checkpoint DDT models to experimental data from multiple human cell lines (nonmalignant and cancer) under different growth conditions and drug treatments. We present the mathematical basis of the DDT modeling approach, compare it to a frequently-used family of models known as exponentially-modified peak functions (EMPF) [58], and describe a custom numerical method that enables parameter estimation for multi-fold convolution models in the face of highly-concentrated distributions. Our analysis shows that in all cases our experimental data is best described by a two-checkpoint model, consistent with the view that the cell cycle has two primary decision points: the commitment to cell division in G1 and the confirmation that the cell is ready to divide in G2 [1]. Furthermore, by comparing model predictions to experimental data quantifying residence times in different segments of the cell cycle, the model predicts a highly variable and growth-factor-sensitive phase that corresponds to a portion of G1. We conclude by comparing our model predictions to competing views of the G1 cell cycle phase proposed in the literature and discuss the potential for interfacing the DDT model with complex bottom-up models of the signaling pathways controlling checkpoint passage.

## Materials and methods

### Cell culture

Human lung adenocarcinoma PC-9 cells, human melanoma A375 cells, benign mammary epithelial MCF10A cells, and MCF10AT1 cells (a variant of MCF10A engineered to stably express V12-Ras that mimics constitutively high levels of growth factor signaling [59]) were cultured as previously described [52, 60]. Numerous publications have demonstrated the effects of Ras transformation by comparing the MCF10AT1 cell to the parental MCF10A cell line [60–63]. All cell lines were engineered to express a fusion protein consisting of histone 2B/monomeric red fluorescent protein (H2B-mRFP) using lentivirus-mediated transduction [60]. A monomeric azami green fluorescent protein in-frame with cDNA of human geminin amino acids 1–110 (fluorescent ubiquitination-based cell cycle indicator, a.k.a. Fucci [64]) was also engineered into A375 cells as a marker of S, G2, or M phases, as described [52]. Treatments to perturb cell cycle progression include erlotinib, a small-molecule inhibitor of the epidermal growth factor receptor (EGFR) tyrosine kinase activity (0.5 *μM*), and cycloheximide (CHX), an antibiotic that inhibits protein synthesis (0.05 *μg*/*μL*). Dimethyl sulfoxide (DMSO) is used as the vehicle control (0.05 %*v*/*v*). Treatment involved replacing medium with complete medium containing the specified agent. DMSO-, erlotinib-, and CHX-treated MCF10A cell data were obtained from a single experiment. MCF10AT1 (untreated) and MCF10A (untreated) were from a separate experiment in which the medium was not changed prior to imaging. No vector control of the V12-Ras-transformed MCF10AT1 cells was available from the group that produced the cell line [59]. Together, these cell lines and conditions represent relevant perturbations of the primary G1 checkpoint.

### Automated fluorescence microscopy imaging

Cells proliferating in culture were imaged as previously described [52, 60]. Briefly, a temperature- and CO_2_-controlled, automated, spinning-disk confocal microscope (Pathway 855, BD Biosciences, Rockville, MD) was used to acquire fluorescence images every 6–30 minutes. Nuclei were enumerated by automated digital image segmentation using the freely available ImageJ program (http://rsb.info.nih.gon/ij/). Individual cells were manually tracked through a series of images, mitotic events were identified, and the number of frames between two successive mitoses was used to determine the IMT [52, 60]. The time of each mitotic event and the IMT value for each completed cell cycle were extracted. In cells expressing the Fucci marker of cell cycle position [52, 64], the time at which Fucci became detectable was also recorded. Time between the first mitotic event and the time of Fucci detection was considered time spent in G1.

### Data censoring

Individual cell IMT data was subject to multiple steps of censoring to remove bias due to cell death, cells reaching the end of the experiment (EoE) without dividing, cell crowding, and delays in stabilization of the drug effect. Cell death was rare in the experiments considered here (only PC-9 and A375 cells exhibited any cell death; Fig B of S1 File). Thus, cells that died were simply excluded from the analysis. To minimize the effect of the EoE, we calculated the last birth time at which > 96% of cells divided before reaching the EoE. Cells born after this time were excluded from the analysis. Thus, we exclusively examined the dividing cells in asynchronously dividing populations. To account for cell crowding (confluence), we analyzed the correlation between birth time and IMT using the Spearman correlation coefficient [65]. Cells born after the time at which the correlation becomes significant (*p* ≤ 0.01) were excluded from the analysis. Finally, erlotinib-treated MCF10A cells exhibited a period of transient growth at the beginning of the experiment, presumably due to a delay in the stabilization of the drug effect [66]. Thus, cells born < 10 h after drug addition were excluded from the analysis in this case. Complete data sets, including censored data, are shown in Fig B of S1 File.

### Stochastic checkpoint models

**Fig 2:**
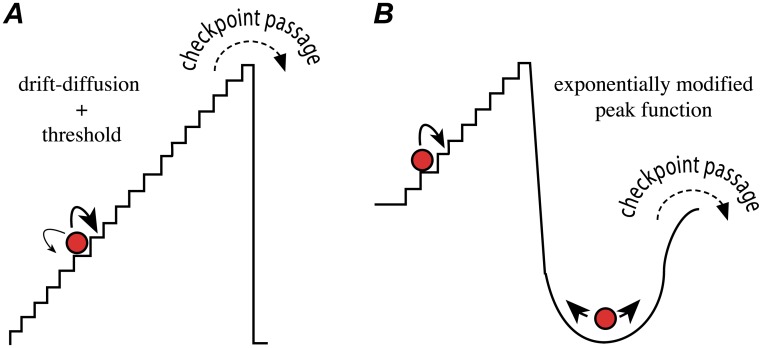
Schematic representations of stochastic cell cycle checkpoint models. (A) The DDT model random variable is depicted as a ball stochastically traversing a staircase. The ball can move both up and down the staircase (diffusion) but the probability of taking a step up is greater than taking a step down (drift; thick arrow vs. thin arrow). Checkpoint passage occurs immediately upon the ball reaching the top of the staircase (threshold), emulating a bistable switch. (B) The EMPF model is depicted as a staircase connected to a basin. Once the ball reaches the top of the staircase it falls into the basin, from which time to escape is exponentially distributed. In contrast to the DDT model, here the ball can only move up the staircase (single arrow). The transition-probability model of Smith and Martin [15] corresponds to the special case in which the time to traverse each step is constant (delayed exponential). The EMG [38, 39] relaxes this assumption, so that the total time to traverse the staircase is Gaussian distributed. See S1 File for a detailed mathematical comparison of the two models.

**Fig 3:**
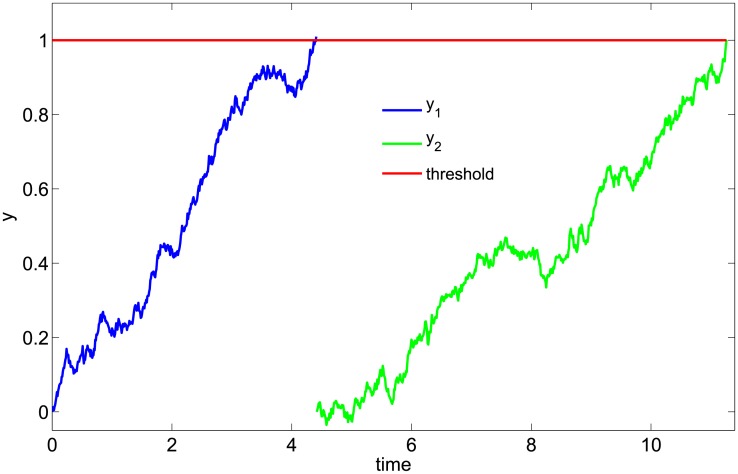
Illustration of a two-checkpoint drift-diffusion+threshold model of the cell cycle. Simulation was performed by numerically solving Eq 1, twice in sequence, using the Euler-Maruyama algorithm [56] with a fixed time step Δ*t* = 0.01. Drift and diffusion constants are *μ*_1_ = *μ*_2_ = 0.2 and *σ*_1_ = *σ*_2_ = 0.1, respectively.

We consider models in which the cell cycle is comprised of *m* ≥ 1 checkpoints with passage times that vary stochastically in length and need not lie at the boundaries between cell cycle phases (see Fig 1). In Ref. [55], we proposed a model in which passage of the *i*-th checkpoint is controlled by a random variable, *y*_*i*_, that represents a “bifurcation parameter” [67] of the underlying biochemical network controlling checkpoint passage. Specifically, we assume that passage of the checkpoint occurs when *y*_*i*_ first reaches a critical threshold value, which triggers an irreversible cell state transition [30, 68]. The temporal evolution of *y*_*i*_ is described by the Itô stochastic differential equation (SDE) [56]

$$\begin{array}{c} dy_{i}\left(t\right)=\mu_{i}dt+\sigma_{i}dW_{t};\;y_{i}\left(0\right)=0, \end{array}$$ (1)

where *μ*_*i*_ and *σ*_*i*_ are the “drift” and “diffusion” constants, respectively, and *W*_*t*_ is a standard Weiner process [56]. Checkpoint passage is assumed to occur when *y*_*i*_(*t*) = 1. We refer to this type of model as a “drift-diffusion+threshold,” or DDT, model. Note that in the most general formulation of Eq 1, the drift and diffusion terms can be functions of time [7, 32]. However, since the growth function describing the mammalian cell cycle is complex and largely unknown [69, 70], we chose the form in Eq 1 because it is the simplest that can capture, without imposing a specific growth function, the aggregate effects of the complex network of molecular interactions underlying checkpoint passage. A schematic representation of a checkpoint in the DDT model is show in Fig 2A; an example simulated time course for a two-checkpoint DDT (DDT2) model is shown in Fig 3. Additional information regarding the mathematical basis of the DDT model is provided in S1 File.

It can be shown that for the stochastic process in Eq 1, the time *t* at which *y*_*i*_(*t*) = 1, i.e., the checkpoint passage time, follows an inverse Gaussian (or Wald) distribution [71, 72],

$$\begin{array}{c} f\left(t;\mu_{i},\sigma_{i}\right)=\frac{1}{\sigma_{i}\sqrt{2\pi t^{3}}}\exp(\frac{-{\left(\mu_{i}t-1\right)}^{2}}{2\sigma_{i}^{2}t}), \end{array}$$ (2)

with mean 1/*μ*_*i*_ and variance $\sigma_{i}^{2}/\mu_{i}^{3}$. Thus, for an *m*-checkpoint DDT model, the probability density function for the IMT is an *m*-fold convolution of inverse Gaussian distributions,

$$\begin{array}{c} p(t;\mu_{1},\sigma_{1},\ldots \mu_{m},\sigma_{m})=\\ \int_{0}^{t}\cdots \int_{0}^{t-\sum_{i=1}^{m-2}s_{i}}f_{1}\left(s_{1}\right)\ldots f_{m-1}\left(s_{m-1}\right)\;f_{m}\;\;(t-\sum_{i=1}^{m-1}s_{i})ds_{1}\ldots ds_{m-1}, \end{array}$$ (3)

where we have used the shorthand *f*_*i*_(*x*) ≔ *f*(*x*; *μ*_*i*_, *σ*_*i*_). For given values of {*μ*_1_, *σ*_1_, … *μ*_*m*_, *σ*_*m*_}, we can solve Eq 3 by approximating the convolution as a left-hand Riemann sum [73] (using the Matlab conv function; www.mathworks.com/help/matlab/ref/conv.html). However, numerical challenges arise if one of the density functions in Eq 3 is highly concentrated (i.e., has a very low variance). To handle this situation, we developed a novel algorithm that automatically identifies and replaces highly-concentrated distributions with Dirac delta functions (an infinitely narrow, infinitely dense mathematical construct) [56, 57] before performing the convolution (see “Parameter estimation for convolution models with highly-concentrated distributions” and S1 File for details). Note that because of the commutative property of convolutions [73], it is not possible to define an ordering of the checkpoints. Therefore, we compare the model parameters to experimental data specific to known cell cycle phases to infer an ordering (see “Results”).

An alternative stochastic checkpoint model that has been used to describe IMT distributions is the exponentially-modified Gaussian, or EMG, model [38, 39]. The EMG model is a two-part model that assumes that passage of a checkpoint involves “transit” and “dwell” phases where duration in the former is Gaussian distributed and duration in the latter is exponentially distributed (see Fig 2B). The checkpoint passage time is thus a convolution of the two distributions. The Gaussian part of the EMG has been associated with S, G2, M, and early G1 while the exponential part has been associated with the G1/S checkpoint [38]. The EMG actually belongs to a larger family of models known as exponentially-modified peak function, or EMPF, models [58] that includes the “transition probability” model of Smith and Martin [15] (delayed exponential, i.e., deterministic transit time) and the exponentially-modified gamma model [40]. It is important to note that because of the exponential dwell time, EMPF models do not describe an ultra-sensitive, switch-like transition mechanism. Given the experimental evidence that checkpoint passage occurs in a switch-like manner [26, 33, 34, 53, 54], we believe that the DDT model is a more accurate description of the biological process of checkpoint passage. A detailed mathematical comparison of the DDT and EMPF modeling approaches is presented in S1 File.

### Parameter estimation for convolution models with highly-concentrated distributions

Best-fit parameter values for all models are obtained using maximum likelihood estimation (MLE) [74]. Let *θ* denote a vector of parameters and *p*(*t*; *θ*) a probability density function being fit to *n* experimental data points {*t*_1_ … *t*_*n*_}, *t*_*i*_ ∈ **R**. The likelihood function is defined as

$$\begin{array}{c} L\left(\theta ;t\right)=\prod_{i=1}^{n}p\left(t_{i};\theta \right). \end{array}$$ (4)

The maximum likelihood estimator, $\hat{\theta }$, is the value of *θ* that maximizes Eq 4. For the DDT models, the density function *p*(*t*; *θ*) is given by the convolution Eq 3. However, numerical inaccuracies can arise in evaluating Eq 3 if one (or more) of the inverse Gaussians (Eq 2) within the integral is highly concentrated. In such cases, the maximum likelihood estimation routine (here we use the Matlab mle function; www.mathworks.com/help/stats/mle.html) can return erroneous answers or fail to converge.

To overcome this problem, we developed a novel, adaptive algorithm for identifying and replacing highly-concentrated distributions with Dirac delta functions [56, 57]. The Dirac delta function *δ*_*τ*_(*s*) can be thought of as an infinitely dense distribution at the point *s* = *τ* ∈ **R**. It is defined mathematically via the following two relations,

$$\begin{array}{cc} & \delta_{\tau }\left(s\right)=0\;\;\;for\;\;\;s\neq \tau , \end{array}$$ (5)

$$\begin{array}{cc} & \int_{-\infty }^{\infty }\delta_{\tau }\left(s\right)ds=1, \end{array}$$ (6)

and has the following property [57],

$$\begin{array}{c} \int_{s_{1}}^{s_{2}}\delta_{\tau }\left(s\right)f\left(s\right)ds=f\left(\tau \right)\;\;\;for\;\;\;\tau \in \left(s_{1},s_{2}\right). \end{array}$$ (7)

If *h* is a highly-concentrated distribution, and both *h* and *f* are zero for *t* < 0, by Eq 7 the convolution $\int_{0}^{t}h\left(s\right)f\left(t-s\right)ds$ can be approximated as

$$\begin{array}{c} \int_{0}^{t}\delta_{\tau }\left(s\right)f\left(t-s\right)ds=f\left(t-\tau \right)\;\;\;for\;\;\;\tau \in \left(0,t\right), \end{array}$$ (8)

where we have set *s*_1_ = 0 and *s*_2_ = *t*. For the DDT models, this means that a two-fold convolution of an inverse Gaussian (Eq 2) with a highly-concentrated inverse Gaussian can be approximated as

$$\begin{array}{c} f\left(t-\tau ;\mu_{i},\sigma_{i}\right)=\frac{1}{\sigma_{i}\sqrt{2\pi {\left(t-\tau \right)}^{3}}}\exp(\frac{-{\left(\mu_{i}\left(t-\tau \right)-1\right)}^{2}}{2\sigma_{i}^{2}\left(t-\tau \right)}), \end{array}$$ (9)

i.e., a translated inverse Gaussian. Doing so improves the accuracy and efficiency of MLE parameter estimation because it avoids the error-prone and computationally expensive step of numerically integrating over highly-concentrated distributions. Note that since an *m*-fold convolution can be solved as multiple, sequential two-fold convolutions, this approach is easily generalized to higher-order DDT models with one or more highly-concentrated distributions. Our adaptive algorithm (outlined in S1 File) identifies theoretical conditions under which a highly-concentrated distribution can be safely substituted by a Dirac delta function, based on the width of the distribution and an estimate of the point-wise error in evaluating the convolution Eq 3 using the approximation. A mathematical derivation of the error bounds used to make this determination are provided in S1 File. A software implementation is freely available at https://github.com/rnleander/DDT_cell_cycle.

### Model selection

To discriminate between candidate cell cycle models (one-, two-, and three-checkpoint DDT models and the EMG model), we use the Akaike information criterion (AIC), an information theoretic approach that estimates the information loss (Kullback-Leibler divergence) when using a model to describe experimental data [75]. We chose the AIC as a model selection metric because it is well established theoretically, widely used in systems biology, easy to employ, and does not require prior knowledge like Bayesian-based methods [76]. The key feature of the AIC is that it mitigates against model overfitting by including a penalty term based on the number of model parameters, i.e., even if a model gives a tighter fit to experimental data, the AIC may prefer a different candidate model if it achieves a comparable fit with fewer parameters. In this work, each *m*-checkpoint DDT model has 2*m* parameters (*μ* and *σ* for each checkpoint) and the EMG model has three parameters (the rate parameter of the exponential and the mean and variance of the Gaussian).

To guard against small-sample effects, we use a modified form of the AIC known as the “AIC with correction for finite size” (AICc) [75]. For the *i*-th model, we calculate

$$\begin{array}{c} {\text{AICc}}_{i}=-2\ln L_{i}\left(\theta ;t\right)+2k_{i}+\frac{2k_{i}\left(k_{i}+1\right)}{n-k_{i}-1}, \end{array}$$ (10)

where *L*_*i*_(*θ*; *t*) is the optimized value of the likelihood function (Eq 4), *k*_*i*_ is the number of parameters, and *n* is the number of data points. Note that the second term on the right-hand side is the “penalty” and the third term is the “correction,” which goes to zero as *n* → ∞. The preferred candidate model is that with the minimum AICc value. Model selection is further aided by the quantity [77]

$$\begin{array}{c} {\text{AICp}}_{i}=\exp(\frac{\min_{j}\left\{{\text{AICc}}_{j}\right\}-{\text{AICc}}_{i}}{2}), \end{array}$$ (11)

which represents an evidence ratio. Note that the preferred model always has an AICp value equal to 1. The AICp can also be used to compare models pairwise, i.e., model *j* can be assumed to be superior to model *i* if the ratio AICp_*i*_/AICp_*j*_ ≪ 1 [77].

### Parameter variability and hypothesis testing

To estimate variability in the values of model parameters, we used bootstrapping, a popular Monte Carlo method for approximating confidence intervals and performing hypothesis tests [78]. For each experimental IMT data set, we sampled the data with replacement to generate 1000 resampled data sets, each the same size as the original data set. For each model, we then performed MLE on each resampled data set to generate an ensemble of model parameter sets. Joint distributions of parameters (*μ* and *σ*) can then visualized as three-dimensional histograms. We also performed hypothesis testing using a two-sample bootstrapping scheme [74]. In this approach, two experimental data sets are combined and sampled with replacement to generate two resampled data sets, one the same size as one of the original data sets and one the same size as the other original data set. MLE is then performed on each resampled data set to obtain best-fit parameter values. For any parameter *θ*, the difference, Δ*θ*′, between the values obtained from the two resampled data sets is recorded. The entire procedure is repeated *N* = 1000 times and each value of Δ*θ*′ is compared against $\Delta \hat{\theta }$, the difference between the best-fit parameter values obtained from the original data sets. We then test the null hypothesis that parameter values for the two data sets are equal by calculating the proportion of samples for which Δ*θ*′ is more extreme than $\Delta \hat{\theta }$, i.e., the p-value

$$\begin{array}{c} p_{\theta }=\frac{1}{N}\sum_{i=1}^{N}1(\Delta \theta_{i}^{\prime }-\Delta \hat{\theta }) \end{array}$$ (12)

if $\Delta \hat{\theta }>0$ or

$$\begin{array}{c} p_{\theta }=\frac{1}{N}\sum_{i=1}^{N}1(\Delta \hat{\theta }-\Delta \theta_{i}^{\prime }) \end{array}$$ (13)

if $\Delta \hat{\theta }<0$. Here, 1(x) is the unit step function that returns 1 for *x* ≥ 0 and 0 otherwise.

## Results

### Modeling predicts two primary cell cycle checkpoints

**Fig 4:**
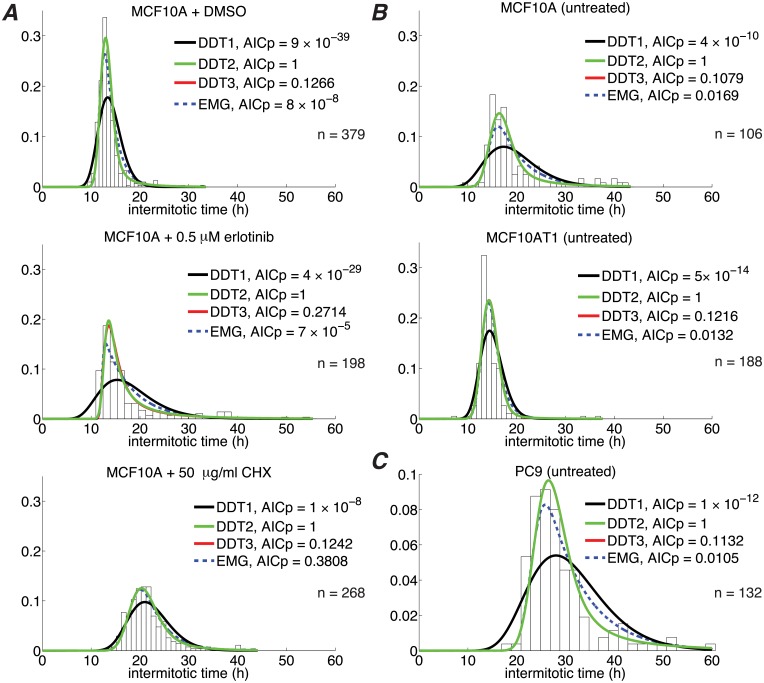
A two-checkpoint drift-diffusion+threshold (DDT2) model best describes IMT variability under numerous conditions. (A), (B), and (C) correspond to independent experiments (with slightly different culture conditions; see “Cell culture”). Note that in most cases the DDT3 curve is obscured by the DDT2 curve. *n*: number of experimental IMT measurements.

We fit the EMG and one-, two-, and three-checkpoint DDT models (DDT1, DDT2, and DDT3, respectively) to experimental IMT data for six different cell line/growth condition combinations (Fig 4). In all cases, the DDT2 model is theoretically preferred, based on the Akaike information criterion (Eq 11). The DDT3 model provides slightly better fits in each case (not visually apparent in Fig 4; see Table A of S1 File) but is penalized for having two additional parameters. However, the DDT3 model cannot be entirely ruled out in any case since all ratios of AICp values with respect to DDT2 are > 0.1 (see “Parameter estimation and model selection”). The EMG model also cannot be entirely ruled out in one case (MCF10A + 50 *μ*g/ml CHX). Nonetheless, the weight of the evidence points to the DDT2 model as being best able to describe the experimentally-observed IMT variability under the growth conditions and perturbations considered here. This result is significant in that it emerges from our modeling approach, rather than being explicitly imposed, and is consistent with the traditional view that the cell cycle has two primary steps, each regulated by a checkpoint: the commitment to cell division prior to cells entering S phase and the process of cell division in mitosis [1].

### DDT models predict a checkpoint with highly variable passage times

**Fig 5:**
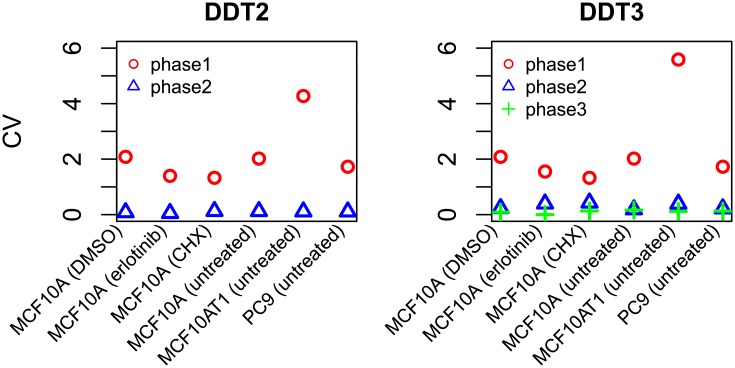
A highly-variable cell cycle phase is preserved between multi-checkpoint DDT models. Relative variability in checkpoint passage times, as quantified by the coefficient of variation (CV), is shown for each phase of the DDT2 and DDT3 models for all experimental conditions considered. Note that the phase numbers are arbitrary (ordered from largest to smallest CV) and do not reflect their actual order in the cell cycle (see “Stochastic checkpoint models”). Hereafter, the high-variability phase is referred to as “H” and the low-variability phase(s) as “L” (L1 and L2 for DDT3).

We next investigated the relative contributions of each phase of the DDT2 and DDT3 models (the theoretically most likely models) to the total IMT. For each phase, we calculated the mean and standard deviation of the checkpoint passage time (see Eq 2) using the best-fit values of the drift (*μ*) and diffusion (*σ*) parameters (Tables B–D of S1 File). We then calculated the coefficient of variation (CV; the ratio of the standard deviation to the mean) as a measure of variability (Fig 5). For both models, we see that one cell cycle phase is significantly more variable than the other(s). Hereafter, we refer to this high variability phase as “H phase” and to the low variability phase(s) as “L phase” (or L1 and L2 in the case of DDT3). Interestingly, the values of the model parameters associated with H phase are well preserved between the DDT2 and DDT3 models. For example, for untreated MCF10A cells, the maximum likelihood parameter estimates for *μ*_*H*_ and *σ*_*H*_ are identical to two significant digits (*μ*_*H*_ ≈ 0.25 and *σ*_*H*_ ≈ 1.0; Table B of S1 File). The fact that the H phase is preserved between models suggests that this phase corresponds to a well-defined biological process that is an important contributor to IMT variability. Moreover, the fact that the DDT3 model breaks the L phase into two less variable phases is intriguing since we know that the cell cycle contains more than two checkpoints (Fig 1).

### The model-predicted highly variable cell cycle phase is growth factor sensitive

**Fig 6:**
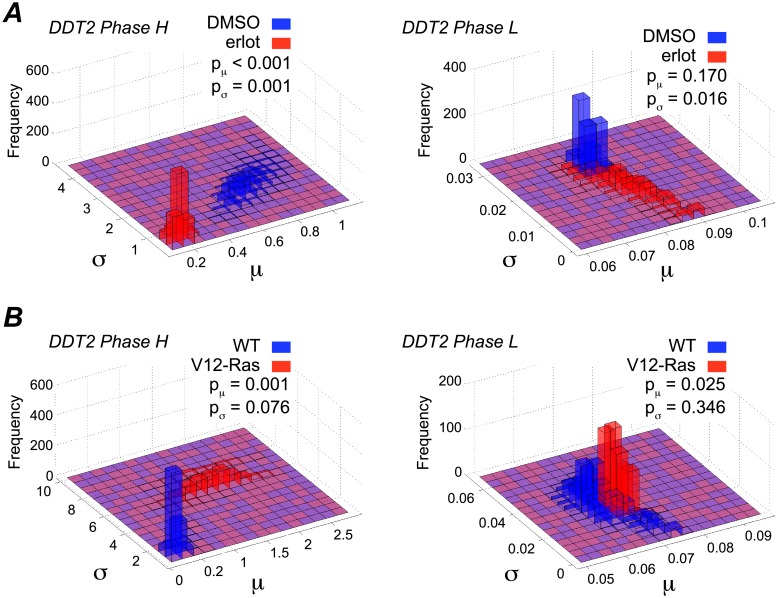
Parameter estimates under growth factor perturbation suggest that the highly-variable (H) phase is growth factor sensitive. (A) MCF10A cells in DMSO control vs. treatment with the EGFR inhibitor erlotinib; (B) MCF10A wild-type (WT) cells vs. MCF10AT1 cells (indicated by V12-Ras). p-values were calculated using a two-sample bootstrapping scheme (see “Parameter estimation and model selection”). Blue signifies unperturbed; red signifies perturbed.

The experimental conditions for the MCF10A cell line include two opposing growth-factor-related perturbations: treatment with erlotinib, a growth factor receptor inhibitor, and stable expression of a mutant form of Ras (V12-Ras) that activates downstream growth factor signaling in the derived MCF10AT1 cell line [59]. To investigate how our modeling approach accounts for these known effects, we compare values of the drift and diffusion parameters, *μ* and *σ*, for both phases of the DDT2 model under perturbed and unperturbed conditions (Fig 6). For both of these perturbations, we see that parameters for H phase are much more sensitive to growth factor perturbations than those for L phase (see “Parameter estimation and model selection”). Specifically, we see that erlotinib treatment significantly decreases *μ*_*H*_ while V12-Ras significantly increases *μ*_*H*_. The mean checkpoint passage time is inversely related to *μ*_*H*_ (Eq 2). Therefore, our model predicts that under erlotinib treatment, time spent in H phase is significantly increased (∼4×) relative to control while stable expression of growth factor (V12-Ras) significantly decreases time spent in H phase (> 4×; Table B of S1 File). Conversely, the model predicts that growth factor perturbations have a much smaller (< 10%) effect on the parameters for L phase (Fig 6; Tables B and C of S1 File). Interestingly, the protein synthesis inhibitor CHX is predicted to lengthen the time spent in both cell cycle phases by a similar amount (∼2×; Fig D and Tables B and C of S1 File). This indicates that the limited effect of increased growth factor on L phase is not simply due to general unresponsiveness. Taken together, our results strongly suggest that the model-predicted H phase is very sensitive to variations in growth factor signaling.

### The highly-variable, growth-factor-sensitive phase correlates with G1

**Fig 7:**
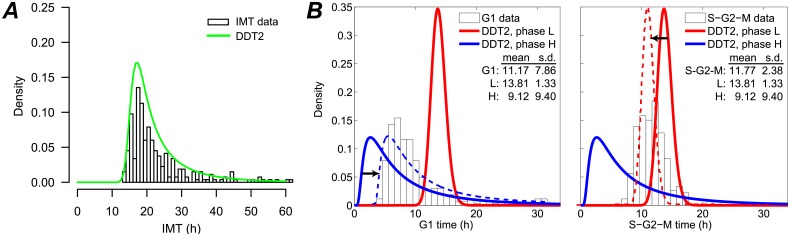
Model-predicted H and L phases correlate with experimental G1 and S-G2-M times. Experimental results and model predictions for untreated A375 cells: (A) IMT data (*n* = 266) overlaid by the best-fit DDT2 model; (B) histograms of times spent in G1 (*left*) and combined S, G2, and M (*right*) cell cycle phases were compared to DDT2 model-predicted H and L phases. The H phase most closely correlates with G1, while the L phase correlates with S-G2-M. Dashed lines indicate distributions shifted (∼3.5 h, indicated by arrows) to best describe the G1 data.

In order to ascertain whether our model-predicted H and L cell cycle phases can be associated with known cell cycle phases, we compared DDT2 model-predicted checkpoint passage time distributions to experimentally-measured times spent in G1 and (collectively) S, G2, and M phases for single melanoma cells (A375, untreated) expressing a live-cell reporter of cell cycle position (Fig 7; see “Automated fluorescence microscopy imaging”). We see that the H phase distribution is very similar in shape to the G1 passage time distribution (with both distributions being highly skewed) while the L phase distribution correlates with the S-G2-M distribution (both distributions are relatively symmetric). The ability of the model to predict a long-tailed distribution of G1 times and a near-symmetric and less variable distribution of S-G2-M times is significant and consistent with experimental evidence that G1 is the primary source of IMT variability [10, 22, 79, 80].

However, the center of the H phase distribution lies to the left of that for the G1 time distribution while the center of the L phase distribution lies to the right of that for the S-G2-M time distribution (Fig 7B). This suggests that the model does not segregate the cell cycle at the phase boundaries. Instead, the model places the G1 checkpoint within the interior of the G1 phase. The remainder of G1, which appears to be of nearly constant duration, is part of a second checkpoint process that spans the remainder of the cell cycle. To better characterize this residual part of G1, we used MLE to shift the H phase distribution such that it optimizes the description of the G1 time data (Fig 7B). We find an optimal translation time of ∼3.5 h. Moreover, if we shift the L phase distribution by the same amount to the left, we see a strong correspondence to the experimental S-G2-M time data, further supporting the idea that a portion of G1 is included in the L phase. This result is in agreement with early published studies suggesting that G1 consists of a highly uniform period of ∼3 h followed by a more variable period that is the primary contributor to IMT variability [22]. However, it is also consistent with more recent studies that associate high variability in cell cycle times to CDK2 activity levels near the beginning of G1 [24, 26] (see “Discussion”).

## Discussion

In progressing through the cell cycle (Fig 1), a cell undergoes discrete and irreversible phenotypic changes [1]. Growing evidence [33, 54] suggests that these changes are controlled by complex molecular networks that act as bistable switches in response to modulating levels of critical biological variables, or “bifurcation parameters” (see below and S1 File). Consistent with this view, here we model the cell cycle as a series of drift-diffusion processes coupled to a threshold (the DDT modeling approach; Figs 2 and 3). An early version of this model was proposed in Ref. [55], where its ability to describe experimental IMT data was demonstrated. Here, we significantly extend this analysis to demonstrate the *predictive* power of the DDT modeling approach. By fitting different variants of the DDT model with one, two, and three checkpoints to experimental IMT data obtained under culture conditions and perturbations designed to modulate the G1 checkpoint, we gain fundamental insights into the structure and control of the cell cycle. Specifically, we find that our data is best described by a model with two checkpoints (Fig 4), one of which has highly variable passage times (Fig 5) and is growth factor sensitive (Fig 6). Furthermore, by comparing model-predicted checkpoint passage times to experimentally-observed cell cycle phase residence times, we are able to associate the highly variable, growth-factor-sensitive phase with a *portion* of G1 (Fig 7).

These findings are broadly consistent with the standard view of the cell cycle as consisting of two primary tasks, the replication of cellular components and the distribution of cellular contents among daughter cells upon division [1]. They are also consistent with multiple studies that report that G1 is composed of multiple portions and is the primary source of variability in IMT [10, 22–24, 26, 27, 79, 80]. There is considerable controversy within this literature, however, regarding the specific structure of G1 and the relative contributions of each portion to IMT variability [23]. Early studies by Zetterberg and co-workers [22, 27] suggest that G1 is comprised of a 3–4 h period of nearly constant duration followed by a highly variable phase that precedes S phase. The early phase is growth factor dependent, in that, removal of growth factors and serum causes cells to arrest into a non-cycling G0 state. Upon re-addition of growth factors, cells can reenter G1 at the same point from which they left. The latter, more variable phase is growth factor *independent* insofar as growth factor withdrawal does not influence G0 arrest. Conversely, more recent work by Meyer and co-workers attributes IMT variability to early G1 and the commitment to divide, specifically linking commitment to increasing CDK2 activity [24, 26]. These authors propose that a fraction of cells are born into a transient G0-like state, characterized by low CDK2 activity, in which they remain for a highly variable (possibly indefinite) length of time before emerging into G1, at which point their CDK2 levels begin to rise. The remaining fraction exhibit increasing CDK2 levels very shortly after mitosis, either by bypassing the G0-like state or emerging from it quickly. This view of G1 as containing an early, highly variable and growth-factor-sensitive portion is also consistent with earlier work by Ho and Tucker [23] and work by Yao et al. [33].

**Fig 8:**
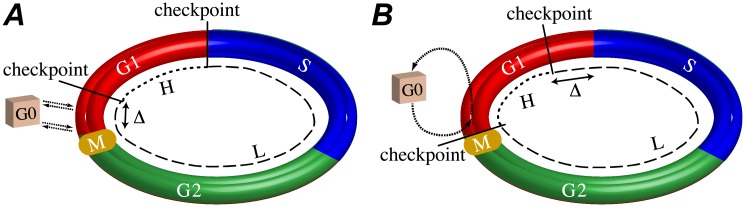
Hypothetical models of G1 checkpoint activity. (A) The H phase begins Δ h *after* the M/G1 phase boundary and ends at the G1/S phase boundary. Cells can enter G0 at any point within the first Δ h of G1, and return at the same position, but not after. This model is consistent with Refs. [22, 27]. (B) The H phase begins at the M/G1 phase boundary and ends Δ h *before* the G1/S phase boundary. Some cells enter into the cell cycle in G0 and reside there for some time before stochastically emerging into G1, which is the source of variability in this phase. This model is consistent with Refs. [23, 24, 26, 33]. Note that Δ ≈ 3.5 h for A375 cells (Fig 7).

In Fig 8, we show schematic illustrations of these two competing views of the structure of G1 and their relation to our model predictions. The model proposed by Zetterberg and co-workers [22, 27] places our H phase at the end of G1, immediately preceding S phase, and the near-constant portion of G1 at the end of L phase (Fig 8A). The H phase is growth factor independent in this view and the checkpoint preceding it is termed the “restriction point.” The model of Meyer and co-workers [24, 26], on the other hand, places the H phase at the beginning of G1 and explains the high variability of this phase in terms of stochastic transitions out of a transient G0-like state (Fig 8B). While we currently cannot eliminate either of these possibilities, our findings tend to support the view of Meyer and co-workers. In particular, our finding that the H phase is growth factor sensitive appears to be in conflict with Zetterberg and co-workers’ assertion that IMT variability emanates primarily from the growth-factor-independent portion of G1 following the restriction point. We cannot say this definitively, however, as their definition of growth factor dependence (see above) and our definition of growth factor sensitivity (increased growth factor signaling decreases time spent in this portion, and vice versa) are not entirely congruent. Furthermore, Zetterberg and co-workers’ model also implies that the DDT model is failing to detect two well-characterized M phase checkpoints [81, 82] (see Fig 1). Again, while this seems unlikely, we cannot rule it out since our experimental approach focused on modulating growth factor signaling, which is known to specifically regulate the G1 checkpoint; other growth-factor-insensitive checkpoints may have been largely inactive and had a negligible influence on cell cycle kinetics [82, 83]. These points emphasize the need for future experiments aimed at modulating different cell cycle checkpoints. For example, paclitaxel-related microtubule inhibitors are known to cause cells to prolong or arrest in mitosis [84] and platinum-based DNA damaging agents cause cells to prolong or arrest in S phase [85]. Coupling these experiments with the DDT modeling approach may allow us to construct over time a more definitive map of the locations of cell cycle checkpoints and resolve this controversy.

As demonstrated here, using the DDT modeling approach as a predictive discovery tool requires considering variants of the model with increasing numbers of checkpoints and applying model selection techniques to identify the best candidate model. A consequence of this approach, however, is that models with “too many” checkpoints will invariably be considered. In such cases, parameter estimation routines tend to accommodate the spurious checkpoints by inserting highly-concentrated distributions into the cell cycle description. For example, in this work, we find that in all cases the DDT3 model differs from the DDT2 model only in a checkpoint with little to no variability (Fig 5; Tables C–D of S1 File). The inclusion of highly-concentrated distributions in the model description poses a significant practical challenge for parameter estimation, i.e., numerical integration techniques can return incorrect solutions or fail to converge. Thus, an additional contribution of this work is the development of an adaptive parameter estimation algorithm that detects and replaces highly-concentrated distributions with Dirac delta functions (see “Parameter estimation for convolution models with highly-concentrated distributions”). The basis of this approach is a mathematical estimation of the error bounds due to substituting a Dirac delta function into the convolution Eq 3. The mathematics is non-trivial (S1 File) and we thus provide an open-source software implementation of the method (https://github.com/rnleander/DDT_cell_cycle). This will facilitate future studies using the DDT modeling approach to explore additional cell cycle checkpoints.

While we have shown that the top-down DDT modeling approach can provide meaningful insights into the inner workings of the cell cycle, the level of molecular detail that can be gleaned from this approach alone is limited. To uncover details of the complex biochemical networks that underlie checkpoint passage and IMT variability—necessary for developing novel therapies for diseases characterized by cell cycle dysregulation (e.g., cancer)—future work will aim to connect DDT models to detailed, bottom-up kinetic models of cell cycle regulation. The key to accomplishing this is to first assign biological identities to the random variables, *y*_*i*_, defined in the DDT model (Eq 1) and then build detailed biochemical models of the complex molecular networks that regulate those variables in an iterative cycle of model building, experimentation, and refinement. For example, as described above, the random variable for passage of the G1 checkpoint may be CDK2 activity [24, 26]. In bacteria, Dinner, Scherer and co-workers have shown that cell size and septal length correlates with IMT [7, 32]. Mathematically, quantities such as these are potential bifurcation parameters of an underlying cell cycle control system, e.g., parameters that determine changes in the number or stability of the equilibrium states of a dynamical system [67, 68, 86]. For complex systems, bifurcation parameters may be composite functions of numerous such variables [68].

Once hypotheses about the identities of the model random variables have been made, detailed biochemical models can be constructed through a combination of literature review and experimentation. The entire process is likely to entail multiple iterations of refinement. In S1 File, we provide a simple example illustrating how hypotheses about a random variable’s identity might be formed. We use a previously proposed model of the biochemical processes controlling passage of the restriction point [68] and show how *y*_*i*_ can be related to cyclin D concentration, *d*, via the relation $\frac{\log\left(d\right)-\log\left(d_{0}\right)}{\log\left(d^{*}\right)\log\left(d_{0}\right)}$, where *d*_0_ is the basal concentration and d* is the threshold concentration above which checkpoint passage occurs. The DDT modeling approach thus provides a simple framework for connecting variability in checkpoint passage times to detailed biochemical models and provides a powerful means to uncover novel details of the complex molecular networks that underlie cell cycle progression.

## Supporting information

S1 File Supplementary information. Comparison of the DDT and EMPF modeling approaches; connecting the DDT model to biological mechanism; numerical methods; supplementary references; supplementary Figs A–D; supplementary Tables A–E.
(PDF)
