# Supplementary material for: A drift-diffusion checkpoint model predicts a highly variable and growth-factor-sensitive portion of the cell cycle G1 phase
Source: PLoS One. 2018 Feb 12;13(2):e0192087. doi: 10.1371/journal.pone.0192087 (PMC5809023; doi:10.1371/journal.pone.0192087)
Supplement: S1 File — Comparison of the DDT and EMPF modeling approaches; connecting the DDT model to biological mechanism; numerical methods; supplementary references; supplementary Figs A–D; supplementary Tables A–E. (PDF) [file pone.0192087.s001.pdf]

SUPPLEMENTARY INFORMATION

A Drift-Diffusion Checkpoint Model Predicts a Highly Variable and Growth-Factor-Sensitive Portion of the Cell Cycle G1 Phase

Zack W. Jones[1◑#a], Rachel Leander[1◑*], Vito Quaranta[2], Leonard A. Harris[2‡], Darren R. Tyson[2‡]

1 Department of Mathematical Sciences, Middle Tennessee State University, Murfreesboro, TN 37132
2 Department of Biochemistry, Vanderbilt University School of Medicine, Nashville, TN 37232

◑These authors contributed equally to this work.
‡These authors also contributed equally to this work.
#a Current Address: Department of Pharmaceutical Sciences, St. Jude Children's Research Hospital, Memphis, TN 38105
*rachel.leander@mtsu.edu

Contents

1 Comparison of the DDT and EMPF modeling approaches **2**
1.1 Exponentially modified peak functions 2
1.2 The drift-diffusion+threshold modeling approach 3

2 Connecting the DDT model to biological mechanism **4**

3 Numerical methods **4**
3.1 Avoiding local maxima and minimizing numerical errors in MLE 5
3.2 Criteria for approximating highly-concentrated distributions as Dirac delta functions 5
3.3 Outline of the algorithm 8
3.4 Validation of the numerical method 10

Supplementary References **10**

Supplementary Figures **12**

Supplementary Tables **15**

1 Comparison of the DDT and EMPF modeling approaches

Here we consider two different approaches to modeling a stochastic cell cycle, the drift-diffusion+threshold (DDT) approach and exponentially modified peak function (EMPF) approach. These approaches are similar in that they both treat the cell cycle as a sequence of stochastic processes. However, there are also important distinctions between them, which we feel make the DDT approach more suitable for describing the stochastic cell cycle as it is controlled by a cell's molecular mitotic state. Such a description is desirable because it coincides with our current understanding of cellular decision making processes and has the potential to provide mechanistic biological insights into cell cycle control.

1.1 Exponentially modified peak functions

In this modeling approach the cell cycle is divided into parts which occur in sequence. Some parts of the cell cycle may be of constant duration or have normally distributed durations while others may be more highly stochastic. As described in the main text, these more stochastic cell cycle parts may be considered as the time a cell waits to pass a checkpoint having obtained an admissible state (e.g. having obtained a sufficient size). Exit from these more variable cell cycle parts is determined by a random variable in such a way that the time spent in these parts is either exponentially or gamma distributed. In evaluating this model, it is instructive to consider the assumptions under which it is derived. Following [1,2], suppose that over a short interval $[t, t + \Delta t]$ the probability of observing a certain "cell cycle event" is $r\Delta t + o(t)$, where $\lim_{\Delta t \to 0} \frac{o(\Delta t)}{\Delta t} = 0$, while the probability of not observing a cell cycle event is $1 - r\Delta t + o(t)$. If we let $p_i(t)$ denote that probability that we have observed i cell cycle events at time t, given we had observed no events at $t = 0$, we find that for $i \geq 1$,

$$p_i(t + \Delta t) = p_{i-1}(t)(r\Delta t + o(t)) + p_i(t)(1 - r\Delta t + o(t)),$$

and,

$$p_0(t + \Delta t) = p_0(t)(1 - r\Delta t + o(t)).$$

Rearranging these equations, dividing by Δt, and taking the limit as Δt approaches 0, we have that for $i \geq 1$,

$$p_i'(t) = r(p_i(t) - p_{i-1}(t)),$$

and $p_0'(t) = rp_0(t)$. Setting $p_0(0) = 1$ and $p_i(0) = 0$, for $i \geq 1$, i.e. assuming that initially no cell cycle events have been observed, we solve these equations recursively to find that the number of events observed up to time t is Poisson distributed with parameter rt. It follows that the time to observe one event is exponentially distributed while the time to observe multiple events is gamma distributed [2]. In summary, if we assume that exit from a part of the cell cycle coincides with the observation of the kth cell cycle event, the distribution of time spent in that part of the cell cycle will be exponentially or gamma distributed in case $k = 1$ or $k > 1$, respectively.

While this model is a reasonable first approximation of checkpoint passage, its underlying control variable, the cell cycle event, is too limited to provide an accurate description of a cell's molecular mitotic state. The difficulty is two-fold. First, since the observance of cell cycle events cannot be undone, the number of events observed is an inflexible representation of a cell's state. These events, for example, cannot represent the concentration of a cellular protein because a concentration can both increase and decrease. In addition, according to this model a cell's probability of experiencing a single event over an interval of length Δt is a constant, $r\Delta t$, whereas it is desirable that this probability should vary through time, i.e. with the cell's dynamic state [3–5]. Second, this model provides a very limited range of variability because the coefficient of variation for the gamma distribution with $k \geq 1$ (i.e. with one or more events), is bounded above by 1 and will converge to zero as k (the number of events) grows large [6]. Meanwhile our data suggest that a variable

portion of G1 has a coefficient of variation that is greater than one. In summary, the EMPF modeling approach does not describe cell cycle time as it is controlled by a cell's molecular state because the model's underlying timer, the number of cell cycle events observed, is monotone increasing and integer-valued, while the cell's molecular mitotic state is dynamic and almost continuous. Moreover, this model of the cell cycle may not be flexible enough to capture high levels of variability that characterize the most variable cell cycle parts.

1.2 The drift-diffusion+threshold modeling approach

Recent research suggests that cellular decisions are governed by proteins thresholds [7–9]. As such, we conceptualize a cell's molecular mitotic state with respect to a given checkpoint as being represented by a real-valued function, y, of multiple such proteins $p_1, p_2, \ldots p_m$. Let's call y the molecular mitotic state function. Granted, this representation is necessarily a simplification (a cell's full state is multidimensional), however, it is a reasonable first approximation that is in line with current thinking about cell cycle control. For example, in [7], a scalar, the ratio of cyclinD1 activity to that of p21 activity, was proposed to act as a cell cycle timer. Moreover, evidence suggests that cellular decisions occur when protein-related functions obtain critical threshold values (e.g. cyclinD1:p21 =1). Hence y is a function that projects a cell's full molecular state onto the real line in such a way that cellular states which correspond to checkpoint passage map to a single critical value. This final point incorporates additional realism into the model because it allows the full molecular state of a cell at the time of checkpoint passage to vary. That is, the set of all molecular states that correspond to checkpoint passage is a level curve of the molecular mitotic state function, not a single point in the cell's full state space.

The DDT model is based on the preceding considerations. Like the EMPF, its mathematical development begins with a discrete model, but now we assume the random variable that captures a cell's molecular mitotic state can both increase and decrease over a short interval of time. Following [1,10], suppose that over a short interval of time $[t, t + \Delta t]$, y may increases by Δy with probability $B\Delta t = (\frac{c}{2\Delta y^2} + \frac{b}{\Delta y})\Delta t$, or decrease by Δy with probability $D\Delta t = (\frac{c}{2\Delta y^2} + \frac{d}{\Delta y})\Delta t$, where $\Delta y^2 > \Delta t$. Letting $y_i = i\Delta y$ and $p(y_i, t)$ denote the probability $y(t) = y_i$, we have that

$$p(y_i, t + \Delta t) = p(y_{i-1}, t)B + p(y_{i+1}, t)D + (1 - B - D)p(y_i, t).$$

Taylor expanding the left hand side in terms of t, the right hand side in terms of y, and dividing the result by Δt, we find that

$$\frac{\partial p}{\partial t}(y,t) + \frac{o(\Delta t)}{\Delta t} = (D - B)\Delta y \frac{\partial p}{\partial y}(y,t) + (D + B)\frac{\Delta y^2}{2}\frac{\partial^2 p}{\partial y^2}(y,t) + o(\Delta y^2)\frac{(B + D)}{\Delta t}.$$

Now letting Δy and Δt approach zero, we arrive at a partial differential equation for the probability density of molecular mitotic states:

$$\frac{\partial p}{\partial t}(x,t) = -(b - d)\frac{\partial p}{\partial x}(x,t) + \frac{c}{2}\frac{\partial^2 p}{\partial x^2}(x,t).$$

It can be shown [11] that this probability density corresponds to the following Itô stochastic differential equation:

$$dy(t) = \mu dt + \sigma dW(t), \tag{A}$$

where $\mu = (b - d)$, $\sigma^2 = c$, and $W(t)$ is a Wiener process [1]. Importantly, in passing to the limit, we have let both the time step and increment between adjacent states grow small so that we arrive at a description of the cell's molecular mitotic state as a continuous-valued, continuous random variable that can both progress toward and digress away from a threshold level, the attainment of which corresponds to the checkpoint

passage. Hence, in addition to yielding a description of a cell's molecular mitotic state, the DDT modeling approach, like the EMPF approach, yields transition probabilities for cell cycle checkpoints. These probabilities, however, now vary with the underlying molecular mitotic state.

2 Connecting the DDT model to biological mechanism

In order to unlock the detailed biochemical mechanisms that underlie checkpoint passage and IMT variability, which is crucial for developing novel therapies for diseases characterized by cell cycle dysregulation, such as cancer, we must connect the DDT modeling approach with protein dynamics. The key to accomplishing this will be to assign a biological identity to the model's random variables and then test this assignment in an iterative cycle of model building, experimentation, and refinement. We employ a model of the restriction point from Ref. [12] to illustrate how hypotheses about the random variables' identities might be formed. This model describes the interplay between the transcription factor E2F, which promotes the G1/S transition, and its repressor, the retinoblastoma protein (Rb).

It includes a positive feedback loop, whereby E2F promotes the inhibition of its own inhibitor, resulting in bistability [12], i.e., an abrupt system change occurs when a bifurcation parameter, the concentration of a starter kinase, reaches a critical threshold value. We consider cyclin D as a proxy for the starter kinase since cyclin D promotes the activity of its kinase partners (CDK4 and CDK6) [13] and accumulation of cyclin D is essential for passage of the restriction point [14]. In the DDT model (Eq. A), the random variable y_h is normally distributed at all points in time [1, 15]. Since cellular protein concentrations are, on the other hand, generally log-normally distributed [16], we relate cyclin D concentration to y_h through the following equation

$$y_h = \frac{\log(z) - \log(z_0)}{\log(z^*)\log(z_0)}, \tag{B}$$

where z is the cyclin D concentration, z_0 is the basal concentration, and z^* is the threshold value. Note that this equation implies the z is log-normally distributed, as desired. Solving Eq. (B) for z, we derive a stochastic differential equation for z. By Itô's formula [1]

$$dz(t) = \left(\mu C z + \frac{1}{2}\sigma^2 C^2 z\right) dt + \sigma C z \, dW_t, \tag{C}$$

with $C := \ln \frac{z^*}{z_0}$. This equation is of interest because it can represent the dynamics of a cellular protein, or other biological quantity, that is subject to positive feedback. It is important to note, however, that many different stochastic differential equations, in fact any equation that corresponds to a twice continuously differential transformation of y, can be considered as describing a random variable which controls a checkpoint process under the DDT model.

The above transformation can also be used to parameterize the DDT model in order to test hypotheses about the identity of the model's random variable. This is because the basal and threshold levels of the transformed random variable together with the distribution of checkpoint passage times determine the transformed variable's dynamics. Indeed the dynamics of the transformed random variable are sensitive to the threshold that corresponds to checkpoint passage (see Fig. **A**).

In summary, we see that the DDT model provides a highly flexible description of IMT variability that is compatible with mechanistic models and data alike.

3 Numerical methods

The numerical identification of maximum likelihood parameter estimates for convolution models involves multiple challenges, including the potential for local maxima, numerical error, and discontinuities in the

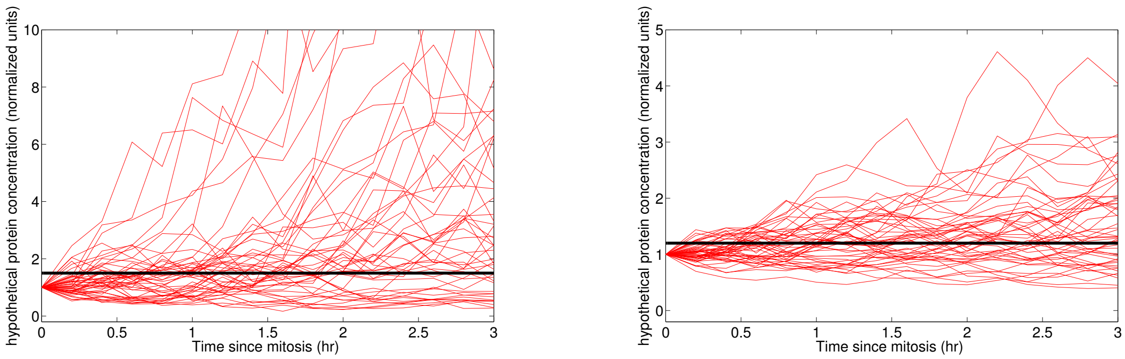

Fig. A. Artificial protein trajectories are generated using the Euler-Maruyama method with a step size of 0.01 h and Eq. (B). Values along the trajectory are plotted in 12 min increments to reflect the limited resolution of the experimental data. The basal level, z_0, is normalized to 1. Trajectories for two threshold values (thick horizontal black lines), z^*, are shown: (*left*) 1.5; (*right*) 1.2.

probability density function on the edge of the parameter space.

3.1 Avoiding local maxima and minimizing numerical errors in MLE

To address the problem of local maxima, we varied the initial parameter guess about parameter choices that match the sample mean and standard deviation. Specifically, for the one-part model (DDT1) we consider all parameter combinations in which the mean and variance of the IMT represent 0.5, 1, 1.5, or 2 times the sample mean and variance. For the two-part models (i.e., the EMG and DDT2) we consider all distinct parameter combinations in which the mean and variance in a part of the cycle represent 0.25, 0.5, or 0.75 times the sample mean or variance of the full IMT. For the three-part model (DDT3), we consider all distinct parameter combinations in which the mean and variance in a part of the cycle represent 0.1 or 0.7 times the sample mean or variance of the full IMT. In forming these distinct parameter combinations, we note that the model is invariant with respect to the ordering of the parts.

To ensure that numerical errors were small, we employed an adaptive method that tracks the error in the likelihood estimation. This error comes from the numerical integration involved in computing the likelihood of the data for the convolution models. Because we approximate the convolution as a left-hand Riemann sum, the error scales with the step size and can be estimated by recalculating the likelihood of the data using a smaller step-size. Thus, for each parameter choice, beginning with a step size of 0.01, we reduce the step size by 50% until two subsequent estimations of the likelihood of the data are within 0.001 of the absolute value of the most recent estimate of the likelihood of the data. That is, our criteria depends on the relative error in the likelihood of the data.

3.2 Criteria for approximating highly-concentrated distributions as Dirac delta functions

The numerical approximation of the model probability density function is a more fundamental challenge, especially for the three-checkpoint (DDT3) model. For both two- and three-checkpoint models (convolution models), the distribution of time spent in a single part of the cell cycle can become highly concentrated, close to a point-mass, or Dirac delta, distribution [17] (probability concentrated at a single point) in the course of parameter estimation. When this is the case, it is exceedingly difficult to get an accurate numerical approximation of the likelihood function. This difficulty is compounded by the fact that for certain initial

guesses and data the maximum likelihood routine converges to such Dirac delta distributions. Highly concentrated distributions can lead to long run times, inaccurate estimates of the likelihood of the data and, in some cases, lack of convergence. We circumvented the computational challenge associated with highly concentrated distributions by approximating these distributions as Dirac delta distributions, thus converting a unwieldy numerical integral to a simple translation. Our criteria for applying this approximation is based on the point-wise error in the approximation.

Suppose that the probability density of IMTs is given by a convolution of two probability density functions g and f, where f is highly concentrated with mean m. If the point-wise error of approximating the convolution of g and f as the convolution of g with Dirac delta distribution centered at m is less than some small ϵ, our method is to approximate f as a Dirac delta distribution ($\epsilon = .01$ in practice). Specifically, we use the standard deviation of f, which for the inverse Gaussian distribution is given by $\frac{\sigma}{\mu^{3/2}}$ [11], to determine if f is concentrated enough to *consider* an approximation. If $\frac{\sigma}{\mu^{3/2}} < 0.01$ h, we estimate the point-wise error of the approximation as follows. First, a small radius, r, is chosen so that over an interval of radius r the change in g is less than $\epsilon/3$. Such an r is determined by first establishing an upper bound, G', on $\{g'(x) : x \in [0, \infty)\}$ and then choosing $r = \frac{\epsilon}{3G'}$. Indeed, with this choice of r and for $|x - y| < r$, it follows from the mean value theorem [18] that there exists c between x and y, such that $|g(y) - g(x)| = g'(c)|x - y| < \epsilon/3$. An upper bound on the error in the approximation is then established according to the value of t. We consider three cases:

Case 1: For $t \geq m + r$, the error in the approximation satisfies

$$\left| \int_0^t f(s)g(t-s)ds - g(t-m) \right| \leq \int_0^{m-r} f(s)g(t-s)ds + \int_{m+r}^{\infty} f(s)g(t-s)ds + \left| \int_{m-r}^{m+r} f(s)g(t-s)ds - g(t-m) \right|. \tag{D}$$

We majorize the first and second terms on the right-hand side of the inequality using an upper bound, G, of $\{g(x) : x \in [0, \infty)\}$, i.e.,

$$\int_0^{m-r} f(s)g(t-s)ds + \int_{m+r}^{\infty} f(s)g(t-s)ds \leq GI, \tag{E}$$

where

$$I = \int_0^{m-r} f(s)ds + \int_{m+r}^{\infty} f(s)ds$$

is small provided that f is concentrated about m. For the the final term to the right of the inequality we use

$$\left| \int_{m-r}^{m+r} f(s)g(t-s)ds - g(t-m) \right| = |(1-I)g(t-y) - g(t-m)| \leq GI + \epsilon/3, \tag{F}$$

where the first equality follows from the mean value theorem for integrals [18] with $m - r < y < m + r$, and the final inequality follows from our choice of r. Thus, with r as above and $GI \leq \epsilon/3$, the point-wise error of the approximation is less than ϵ for $t \geq m + r$.

Case 2: For $t \leq m - r$, the error in the approximation satisfies

$$\left| \int_0^t f(s)g(t-s)ds \right| \leq \int_0^{m-r} f(s)g(t-s)ds \leq GI. \tag{G}$$

Thus, if f is so concentrated that $GI \leq \epsilon/3$, the point-wise error of the approximation is less than ϵ for $t \leq m - r$.

Case 3: To ensure that the error is small for $m - r < t < m + r$, we place an additional constraint on r. In particular, we choose r so that $g(t) < \epsilon/2$ for $t < r$. This is possible because if g is an inverse Gaussian distribution, or a convolution of inverse Gaussian distributions, $\lim_{r \to 0^+} g(r) = 0$ and $g'(r) > 0$ for small r. Thus, for small r and $m - r < t < m + r$ we see that the error satisfies

$$\left| \int_0^t f(s)g(t-s)ds - g(t-m) \right| \leq \int_0^{m-r} f(s)g(t-s)ds + \left| \int_{m-r}^t f(s)g(t-s)ds - g(t-m) \right|, \tag{H}$$

where $g(t - m) = 0$ for $t < m$. Note that by the mean value theorem for integrals there exists y such that $m - r < y < t < m + r$ and

$$\int_{m-r}^t f(s)g(t-s)ds = g(t-y) \int_{m-r}^t f(s)ds.$$

In the subcase that $m + r > t > m$, the final term in Eq. (H) is then bound by $\epsilon/2$ as

$$-g(t-m) \leq g(t-y) \int_{m-r}^t f(s)ds - g(t-m) \leq g(t-y) - g(t-m). \tag{I}$$

The left- and right-hand sides of the inequality are bound in magnitude by $\epsilon/2$ by our choice of r since $0 < t - m < r$ and $|(t - y) - (t - m)| = |m - y| < r$. Similarly, in the subcase $m - r < t \leq m$, the final term in Eq. (H) is bound by $\epsilon/2$ as

$$0 \leq g(t-y) \int_{m-r}^t f(s)ds \leq g(t-y), \tag{J}$$

and the right-hand side of the inequality is bound by $\epsilon/2$ by our choice of r since $0 < t - y < m - (m - r) = r$. Since the first term in Eq. (H) is bound by IG, as before, we see that the error in the approximation is less than ϵ provided $IG < \epsilon/3$.

In summary, if r is small so that (i) $r < \epsilon/G'$ and (ii) $g(t) < \epsilon/2$ for $t < r$, then if f is so concentrated that $GI \leq \epsilon/3$, the point-wise error of the approximation is less than ϵ and we approximate f as a Dirac delta distribution centered at m when computing the likelihood of the data. Otherwise, the likelihood is computed using the full numerical approximation of the convolution of f and g.

In justifying the method described above we used the fact that if g is an inverse Gaussian, i.e., is distributed according to

$$p_i(t; \mu, \sigma) = \frac{1}{\sigma\sqrt{2\pi t^3}} \exp\left(\frac{-(\mu t - 1)^2}{2\sigma^2 t} \right), \tag{K}$$

or a convolution of inverse Gaussian distributions, then $\lim_{t \to 0^+} g(t) = 0$ and $g'(t) > 0$ is increasing for t. Looking at Eq. (K), it is easy to see that $\lim_{t \to 0^+} g(t) = 0$ when g is an inverse Gaussian distribution and in the case that g is a convolution of inverse Gaussian distributions the result follows from the continuity of the inverse Gaussian. Also, by taking the derivative of Eq. (K) with respect to t, it is also easy to see that this distribution is increasing for $t < T$, where T is the value where the distribution takes its mode. To see that a convolution of inverse Gaussian distributions is increasing for small t note that if $g(t) = \int_0^t g_1(s)g_2(t - s)ds$, where g_1 and g_2 are inverse Gaussian distributions, then

$$g'(t) = \int_0^t g_2(s)g_1'(t-s)ds$$

is positive for $t < T_1$, where T_1 is the value where g_1 takes its mode. Similarly

$$g'(t) = \int_0^t g_1(s) g_2'(t-s) ds$$

is positive for $t < T_2$, where T_2 is the value where g_2 takes its mode. Thus, g is increasing for $t < \max\{T_1, T_2\}$, and the claim is established. In implementing our method we must first find $r < T$ so that $g(r) < \epsilon/2$. This is accomplished in the case that g is an inverse Gaussian distribution by evaluating g at decreasing values of $r < T$, where T is the value where g takes its mode. In the case that g is a convolution of inverse Gaussian distributions, g_1 and g_2, r is chosen by evaluating both g_1 and g_2 at decreasing values of $r < T := \min\{T_1, T_2\}$ and choosing r so that at least one of g_1 or g_2 is less than $\epsilon/2$. Indeed, by the mean value theorem for integrals, for $t < T$, $g(t) \leq \min\{g_1(t), g_2(t)\}$. Thus, if $g_i(r) < \epsilon/2$ for some $r < T_i$, then $g(t) \leq g_i(t) < \epsilon/2$ for all $t < r$.

Our numerical method also requires upper bounds G, G', and I. We obtain G as follows. In the case that g is an inverse Gaussian distribution, we calculate $G := max\{g(x)\}$ by evaluating g at its mode, which can be solved for directly. If g is a convolution of two inverse Gaussian distributions, g_1 and g_2, we obtain an upper bound on g as follows. Since

$$g(t) = \int_0^t g_1(s) g_2(t-s) ds \leq G_2 \int_0^\infty g_1(s) ds = G_2, \tag{L}$$

where $G_2 = max\{g_2(x)\}$, and similarly $g(t) \leq G_1 := max\{g_1(x)\}$, we take $G := \min\{G_1, G_2\}$. In order to obtain G', we also consider two cases. In the case that g is an inverse Gaussian distribution, we find $G' = \max\{|\, g'(x)\,|\}$ by evaluating $g'(x)$ at each of its critical points (these points correspond to the roots of a fourth-order polynomial, which are solved for numerically using the MATLAB `roots` function). In the case that g is a convolution of inverse Gaussian distributions, g_1 and g_2, we establish an upper bound on $g'(x)$ as follows. Since

$$|g'(t)| \leq \int_0^t |g_1(s) g_2'(t-s)|\, ds \leq G_2', \tag{M}$$

where $G_2' = \max\{|\, g_2'(x)\,|\}$, and similarly $|g'(t)| \leq G_1$, we take $G' = \min\{G_1', G_2'\}$. In addition, we approximate I as

$$I = \int_0^{m-r} f(s) ds + \int_{m+r}^\infty f(s) ds \approx \int_0^{m-r} f(s) ds + \int_{m+r}^T f(s) ds, \tag{N}$$

where $T = \max\left\{m + r + 1000\sigma/\mu^{3/2}, 100\right\}$ and the integrals are approximated as Riemann sums. In performing this approximation, we take the initial step size as 0.001 and reduce the step size by a factor of 1/2 until the absolute value of the change in the approximation in less than 10^{-4}.

3.3 Outline of the algorithm

The algorithm for determining if a distribution may be approximated as a Dirac delta function, for the purpose of evaluating its convolution with one or more inverse Gaussian distributions, is summarized below. In what follows, let f denote an inverse Gaussian distribution, and let g denote a convolution of one or more inverse Gaussian distributions (which have greater standard deviations than f). Let τ denote the average of f. Let $f * g$ denote the convolution of f and g. Let $sd(f)$ denote the standard deviation of f.

Algorithm 1

Input: $[\mu_1, \sigma_1 \ldots \mu_m, \sigma_m]$: a vector of m drift (μ) and diffusion (σ) parameters.
T: a vector of n intermitotic times (data).
Output: L: The likelihood of the data.

0. If $sd(f) \geq 0.01$, calculate the likelihood, $L = \prod_{i=1}^{n}(f * g)(T_i)$, using numerical integration to compute each factor in the product;
else, determine if $|(f * g)(t) - g(t - \tau)| < \epsilon$, for all $t \in [0, \infty]$, as follows:

1. Determine the maximum value of g', the first derivative of g. This value is used to determine a radius r_1 so that $|g(t_1) - g(t_2)| < \epsilon/3$ for $|t_1 - t_2| < r_1$.
   - To determine r_1 from the maximum of g' we use the mean value theorem (see Sec. 3.2). If g is an inverse Gaussian distribution, the maximum of g' is found by evaluating g' at each of its critical points, which are identified using MATLAB's root finder (`roots.m`). If g is a convolution of inverse Gaussian distributions the maximum is bounded above using these same methods and properties of the inverse Gaussian distribution and the convolution as described in Sec. 3.2.
   - We require $|g(t_1) - g(t_2)| < \epsilon/3$, because this magnitude represents one of three components of the error in Eq. (D) (see also Eq. F).

2. Determine a radius r_2 so that $g(t) < \epsilon/2$ for $t < r_2$.
   - Properties of the inverse Gaussian distribution, as described in Sec. 3.2, are used to design an algorithm for finding such an r_2. Specifically, beginning at $t_0 = \min(r, t_M)$, where t_M is the value where g takes its mode, evaluate $g(t_i)$. If $g(t_i) < \epsilon/2$, set $r_2 = t_1$, else set $t_{i+1} = t_i/2$ and repeat.
   - The value of $g(t)$ for $t < r_2$ is one of two components of the error in Case 3 (see Eqs. H–J).

3. Set $r = \min(r_1, r_2)$ (so that the error estimate will hold in Cases 1–3 of Sec. 3.2).

4. Evaluate $I := 1 - \int_{\tau-r}^{\tau+r} f(s)\, ds$.
   - Numerical integration: Note that by properties of a distribution, we can evaluate this integral by instead integrating over the complement of $[\tau - r, \tau + r]$, this is preferable because f is not concentrated here.
   - Note that IG is a component of the error in Cases 1–3 of Sec. 3.2 (see Eqs. D, G and H).

5. Evaluate $G := \max_{t \in [0,\infty]} g(t)$.
   - The maximum is found by evaluating g at its critical point, if g is an inverse Gaussian distribution. If g is a convolution of inverse Gaussian distributions the maximum is bounded above using similar methods and properties of the inverse Gaussian distribution and the convolution, as described in Sec. 3.2.

6. If $GI < \epsilon/3$, then $|(f * g)(t) - g(t - \tau)| < \epsilon$, approximate $(f * g)(T_i)$ as $g(T_i - \tau)$ when computing the likelihood, $L = \prod_{i=1}^{n}(f * g)(T_i)$;
else, evaluate $L = \prod_{i=1}^{n}(f * g)(T_i)$ using numerical integration to compute each factor in the product.

7. Return L.

Note that in case g itself is a convolution of inverse Gaussian distributions, the above algorithm is executed again in order to evaluate g.

3.4 Validation of the numerical method

In order to test the accuracy of our numerical method, we apply it to synthetic data: for each model, we use the maximum likelihood parameter estimates for MCF10A cells treated with erlotinib to generate data sets of increasing size ($n = 200$, $n = 1000$, and $n = 10\,000$). For a fixed data size, we generate and fit 10 sets of data and compute the average relative error (supplementary Table **E**). For the DDT2 model, the relative error for $n = 200$ is on the order of 10^{-1}–10^{-2}. As the data size increases the error declines modestly, so that the average error at $n = 10\,000$ is typically on the order of 10^{-2}–10^{-3}. The relative errors in the DDT3 parameter estimates also decrease modestly as the sample size increases. Interestingly, the relative errors for the parameter estimates of σ are generally larger than those for μ, reflecting greater uncertainty in the estimates of σ.

References

1. Allen EJ. Modeling with Itô stochastic differential equations. Upper Saddle River, New Jersey: Dordrecht: Springer; 2007.

2. Hogg RV, McKean JW, Craig AT. Introduction to Mathematical Statistics. 7th ed. Boston, Massachusetts: Pearson; 2013.

3. Svetina S, Žekš B. Transition probability model of the cell cycle exhibiting the age-distribution for cells in the indeterministic state of the cell cycle. In: Valleron AJ, MacDonald PDM, editors. Biomathematics and Cell Kinetics. New York: Elsevier/North-Holland Biomedical Press; 1978. p. 71–82.

4. Cooper S. The continuum model: statistical implications. J Theor Biol. 1982;94:783–800.

5. Tyson JJ, Diekmann O. Sloppy size control of the cell division cycle. J Theor Biol. 1986;118:405–426.

6. Mangel M. The Theoretical Biologist's Toolbox: Quantitative Methods for Ecology and Evolutionary Biology. 2nd ed. New York: Cambridge University Press; 2006.

7. Yang HW, Chung M, Kudo T, Meyer T. Competing memories of mitogen and p53 signalling control cell-cycle entry. Nature. 2017;549:404–408.

8. Albeck JG, Burke JM, Aldridge BB, Zhang M, Lauffenburger DA, Sorger PK. Quantitative analysis of pathways controlling extrinsic apoptosis in single cells. Mol Cell. 2008;30:11–25.

9. Yao G, Lee TJ, Mori S, Nevins JR, You L. A bistable Rb–E2F switch underlies the restriction point. Nat Cell Biol. 2008;10:476–482.

10. Nauman EB. Residence time distributions is systems governed by the dispersion equation. Chem Eng Sci. 1981; p. 957–966.

11. Tweedie MCK. Statistical properties of inverse Gaussian distributions I. Ann Math Stat. 1957;28:362–377.

12. Tyson J, Novák B. Irreversible transitions, bistability and checkpoint controls in the eukaryotic cell cycle: a systems-level understanding. In: Marian Walhout AJ, Vidal M, J D, editors. Handbook of Systems Biology. San Diego: Elsevier; 2013. p. 265–285.

13. Diehl J, Cheng M, Roussel M, Sherr C. Glycogen synthase kinase-3β regulates cyclin D1 proteolysis and subcellular localization. Genes Dev. 1998;12:3499–3511.

14. Sherr CJ, Roberts JM. Inhibitors of mammalian G1 cyclin-dependent kinases. Genes Dev. 1995;9:1149–1163.

15. Gillespie DT. Markov Processes: An Introduction for Physical Scientists. Academic Press, Inc.; 1992.

16. Furusawa C, Suzuki T, Kashiwagi A, Yomo T, Kaneko K. Ubiquity of log-normal distributions in intra-cellular reaction dynamics. Biophysics. 2005;1:25–31.

17. Zill DG. Differential equations with Boundary-Value Problems. 9th ed. Boston, MA: Cengage Learning; 2016.

18. Wade WR. An Introduction to Analysis. 4th ed. Upper Saddle River, New Jersey: Prentice Hall, Pearson Education, Inc.; 2010.

Supplementary Figures

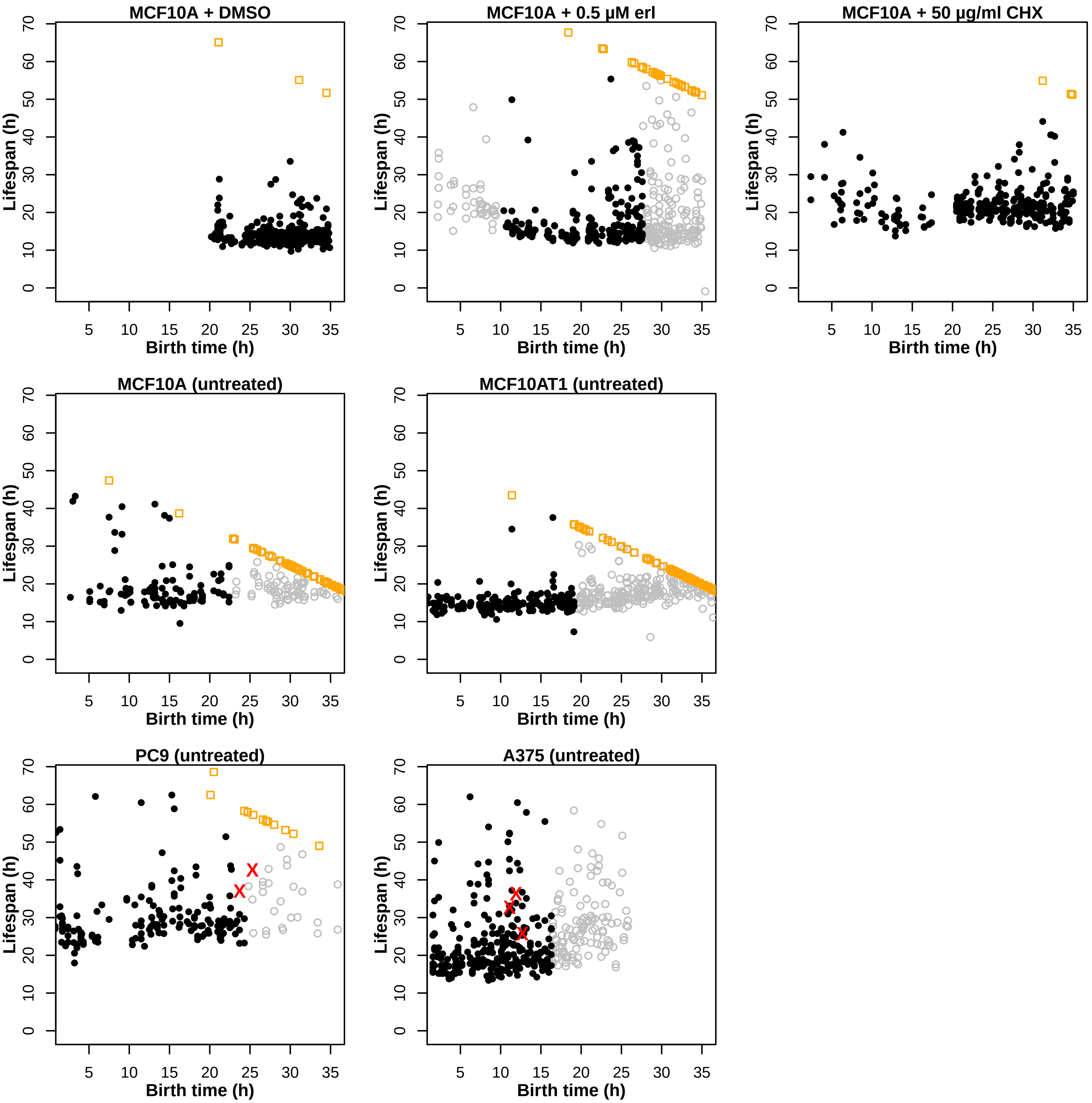

Fig. B. IMT scatter plots illustrating data censoring. Individual cell IMTs vs. birth time (time of the first mitotic event) for seven cell line/growth conditions. (●) Cells included in the analysis; (□) cells excluded because they reached the EoE without dividing; (○) cells excluded either because they were born during the initial drug effect stabilization period ($<$ 10 h in B) or their birth times exceed the last time at which $>$ 96% of cells divided (19.6 h in A; 27.6 h in B); (×) cells excluded because they died during the experiment (only observed in PC9 and A375). Note that the correlation between birth time and IMT (cell crowding) was not significant at the late-time cutoffs in any case, based on Spearman correlation coefficients. Hence, no additional data points were excluded.

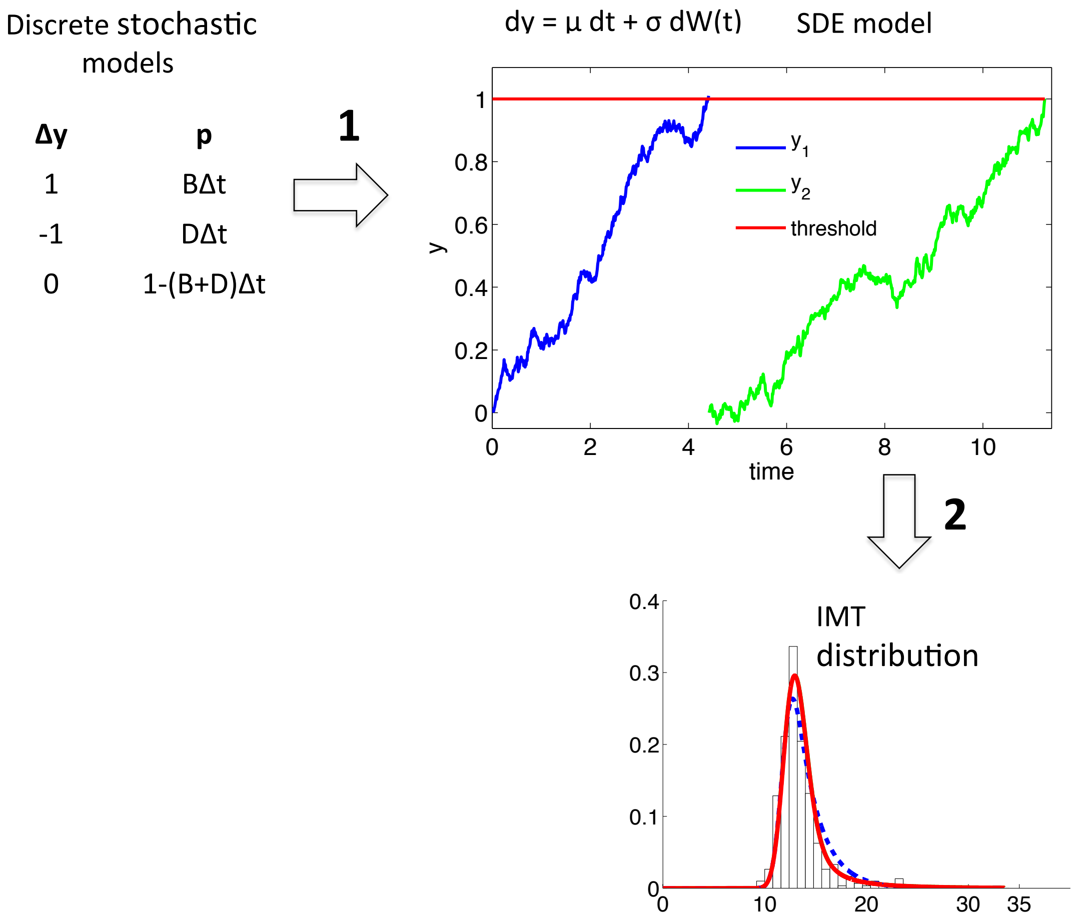

Fig. C. The stochastic process modeling approach. 1. A continuous-stochastic process describing the time evolution of a model random variable is derived from a discrete-stochastic model. **2.** The distribution of checkpoint passage times is found as the distribution of exit times for the continuous model and the checkpoint passage distributions are convolved to yield the IMT distribution.

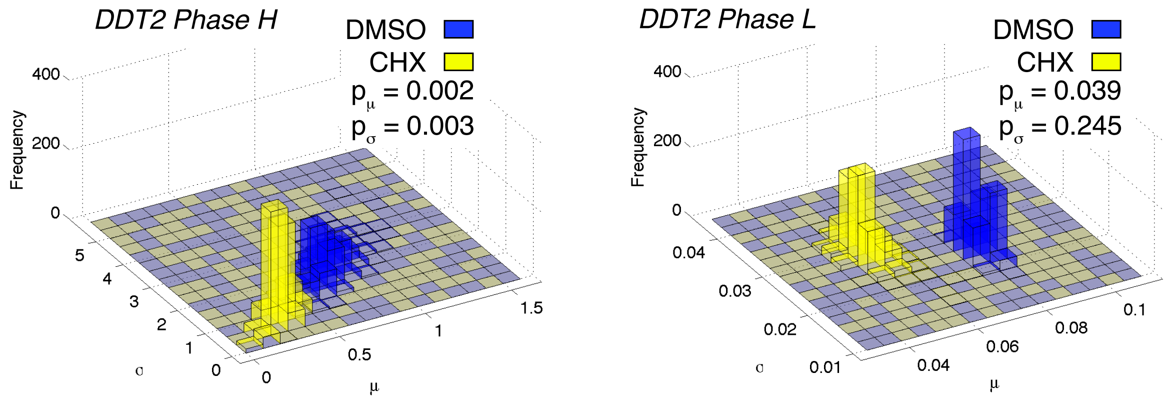

Fig. D. Parameter estimates from both model-predicted phases (H and L) are affected by cycloheximide. IMT data from MCF10A cells treated with 50 μg/ml cycloheximide (CHX; yellow) or vehicle control (DMSO; blue) were fit with the DDT2 model and parameters were obtained by maximum likelihood estimation. Two-sided p-values were obtained using the null hypothesis that the parameter values for cells treated with DMSO are from the same distribution as cells treated with CHX.

Supplementary Tables

Table A. Model comparison. *MLL*: maximum log-likelihood; *AICc*: Akaike information criterion with correction for finite size; *AICp*: pairwise comparsion metric.

	MCF10A, DMSO (n=379)			MCF10A, erlotinib (n=198)		
Model	MLL	AICc	AICp	MLL	AICc	AICp
EMG	-775.211	1 556.486	0	-552.853	1 111.830	0
DDT1	-847.544	1 699.120	0	-609.710	1 223.482	0
DDT2	-757.852	1 523.811	1	-542.296	1 092.800	1
DDT3	-757.859	1 527.944	0.127	-541.484	1 095.408	0.271
	MCF10A, CHX (n=268)			MCF10AT1, untreated (n=188)		
Model	MLL	AICc	AICp	MLL	AICc	AICp
EMG	-742.745	1 491.581	0.381	-396.004	798.138	0.013
DDT1	-761.073	1 526.191	0	-423.389	850.843	0
DDT2	-740.749	1 489.650	1	-390.634	789.487	1
DDT3	-740.750	1 493.822	0.124	-390.618	793.700	0.122
	MCF10A, untreated (n=106)			PC9 (n=132)		
Model	MLL	AICc	AICp	MLL	AICc	AICp
EMG	-304.885	616.005	0.017	-430.149	743.553	0.017
DDT1	-323.614	651.345	0	-454.058	808.992	0
DDT2	-299.723	607.842	1	-424.529	727.288	1
DDT3	-299.723	612.294	0.108	-424.529	731.355	0.108
	A375, untreated (n=266)					
Model	MLL	AICc	AICp			
EMG	-852.060	1 710.212	0.099			
DDT1	-892.381	1 788.808	0			
DDT2	-848.715	1 705.583	1			
DDT3	-848.179	1 708.872	0.193			

Table B. Best-fit maximum likelihood parameter values.

	DDT2				DDT3					
**Data set**	μ_H	σ_H	μ_L	σ_L	μ_H	σ_H	μ_{L1}	σ_{L1}	μ_{L2}	σ_{L2}
MCF10A (DMSO)	0.73	1.8	0.080	0.024	0.73	1.8	0.61	0.18	0.092	0.027
MCF10A (erlotinib)	0.18	0.59	0.082	0.015	0.19	0.67	0.42	0.26	0.10	0.0002
MCF10A (CHX)	0.31	0.74	0.053	0.029	0.319	0.74	0.61	0.34	0.058	0.032
MCF10A (untreated)	0.25	1.0	0.064	0.031	0.25	1.0	0.15	0.07	0.11	0.055
MCF10AT1 (untreated)	1.6	5.4	0.070	0.029	1.9	7.7	0.83	0.36	0.076	0.032
PC9 (untreated)	0.16	0.69	0.040	0.022	0.16	0.69	0.16	0.089	0.054	0.029
A375 (untreated)	0.11	0.34	0.072	0.023	0.11	0.33	0.30	0.20	0.10	0.0004

Table C. DDT2 model-estimated passage time statistics (hours). mean = $1/\mu$; s.d. = $\sqrt{\sigma^2/\mu^3}$.

Data Set	Phase H		Phase L	
	mean	s.d.	mean	s.d.
MCF10A (DMSO)	1.4	2.8	13	1.0
MCF10A (erlotinib)	5.7	7.9	12	0.65
MCF10A (CHX)	3.2	4.3	19	2.4
MCF10A (untreated)	3.9	8.0	16	1.9
MCF10AT1 (untreated)	0.62	2.7	14	1.6
PC9 (untreated)	6.2	11	25	2.7
A375 (untreated)	9.1	9.4	14	1.2

Table D. DDT3 model-estimated passage time statistics (hours). mean = $1/\mu$**;** s.d. = $\sqrt{\sigma^2/\mu^3}$**.**

Data Set	Phase H		Phase L1		Phase L2	
	mean	s.d.	mean	s.d.	mean	s.d.
MCF10A (DMSO)	1.4	2.8	1.60	0.38	11	0.98
MCF10A (erlotinib)	5.3	8.3	2.4	0.94	10	0.0065
MCF10A (CHX)	3.2	4.3	1.6	0.71	17	2.3
MCF10A (untreated)	3.9	8.0	6.7	1.2	8.8	1.4
MCF10AT1 (untreated)	0.53	3.0	1.2	0.47	13	1.5
PC9 (untreated)	6.2	10.7	6.1	1.3	19	2.4
A375 (untreated)	9.2	9.4	3.4	1.2	10	0.012

Table E. Relative error in maximum likelihood parameter estimates.

DDT2 Model				
parameter	true value	($n = 200$)	($n = 1000$)	($n = 10000$)
μ_H	0.1765	0.0897	0.0348	0.0134
σ_H	0.5875	0.1261	0.0510	0.0250
μ_L	0.0891	0.0203	0.0098	0.0052
σ_L	0.0152	0.1781	0.0920	0.0368
**DDT3 Model**				
parameter	true value	($n = 200$)	($n = 1000$)	($n = 10000$)
μ_H	0.1879	0.1349	0.0517	0.0120
σ_H	0.6733	0.2010	0.0982	0.0290
μ_{L1}	0.4182	1.1037	0.1103	0.0519
σ_{L1}	0.2555	0.4278	0.2822	0.0977
μ_{L2}	0.0982	0.0986	0.0560	0.0135
σ_{L2}	0.0002	7.3212	0.5258	0.1501
